# IL1 inhibition in gout—where are we a decade on?

**DOI:** 10.1186/s13075-019-2027-3

**Published:** 2019-11-11

**Authors:** Alexander So

**Affiliations:** Service de Rhumatologie, CHUV and University of Lausanne, Lausanne, Switzerland

Our paper on the use of the IL1 inhibitor anakinra in acute gout was published in *Arthritis Research and Therapy* over 12 years ago [1]. It was a pilot study comprising an extended case series and animal data to validate the concept that IL1 played a pivotal role in the initiation of MSU crystal-induced inflammation. ART was chosen because it does accept and publish proof-of-concept studies rapidly, in comparison with conventional journals. The hypothesis we tested was to assess if IL1 blockade by anakinra, a drug that was already on the market but is licensed only for the treatment of rheumatoid arthritis, would help patients who were unable to receive or had contraindications to conventional drugs used to treat acute gout. The successful result of this uncontrolled study was especially gratifying to Jürg Tschopp (1951–2011), my good friend and colleague who had coined the name inflammasome to describe the newly discovered intracellular “machine” that processed pro-IL1β to mature and secreted IL1β [2]. He showed that a whole range of different triggers was capable of activating the complex, including microcrystals formed by MSU, to initiate inflammation through IL1 (both β and α).

Jürg was particularly concerned that his discovery should have an impact on medical care, and in the heady early days of our success, we dreamt of the clinical impact that targeting the inflammasome could bring about. To a large extent, the initial optimism has been supported by subsequent findings. Greater than nine thousand publications are identified by the keyword inflammasome in PubMed as of the writing of this paper, documenting its participation in a large number of clinical and experimental settings. Further confirmation of the ubiquitous role of inflammation (probably but not exclusively due to inflammasome activation) came from clinical trials of IL1β inhibitors. The CANTOS trial in particular highlighted that IL1-mediated inflammation plays a deleterious role, not only in cardiovascular diseases but also in cancer [3].

Returning to gout, given its efficacy, why is IL1 inhibition not more widely used as an acute treatment? Clinical trials and case series have confirmed that it is effective in acute gout [4] and could be an alternative to NSAIDs, colchicine, and prednisone. The effective blockade of IL1 does carry with it the risk of infection, and although the clinical trials did not show a significant increase in the infection rate, there was nevertheless an increased number of non-fatal infections in the IL1 inhibitor-treated groups. These concerns mean that we should keep IL1 inhibition as a “second-line therapy” for acute gout and be aware of the risks when prescribing such a treatment. In the real world, price concerns and licensing differences (between EMEA and FDA) are also practical hurdles that influence the clinician’s use of such a therapy. The recent randomized controlled study of Janssen, who compared anakinra with conventional “first-line” treatments of acute flare, reassured us that this form of IL1 inhibition was non-inferior to standard therapies and had the same safety profile, with no signal for increased infection [5].

Over the last decade, research on the inflammasome has gathered pace—aiming not only to understand the role of inflammation in particular diseases, but also to dissect the mechanisms that lead to its activation and if this can be modulated for therapeutic ends. Inflammasome inhibitors that act by blocking NLRP3 assembly [6, 7] or by modifying the generation of ROS [8] have shown promise in experimental studies, and the idea that a new class of anti-inflammatories, based on inhibition of the inflammasome, will soon be available to clinicians is no longer unrealistic. If such a treatment were to be developed for acute gout, it could eventually replace NSAIDs and colchicine, as both have safety profiles that are not ideal when they are prescribed to our typical gout patient who presents with multiple co-morbidities. Should these inflammasome inhibitors be effective in gout, there is good reason to believe they will be equally effective in treating inflammation in other inflammasome-mediated diseases (and the list is long and growing).

Finally, our pilot study is an example of how scientific collaborations between basic science (Jürg Tschopp was a biochemist interested in inflammation and immune regulation) and clinicians who have an interest in translational medicine can bring about interesting discoveries that inform our understanding of disease as well as offering new solutions to old medical problems.

## Data Availability

Not applicable.

## Abbreviations

IL1: Interleukin 1
ROS: Reactive oxygen species
